# Weaker Light Response, Lower Stomatal Conductance and Structural Changes in Old Boreal Conifers Implied by a Bayesian Hierarchical Model

**DOI:** 10.3389/fpls.2020.579319

**Published:** 2020-11-06

**Authors:** Che Liu, Teemu Hölttä, Xianglin Tian, Frank Berninger, Annikki Mäkelä

**Affiliations:** ^1^Faculty of Agriculture and Forestry, University of Helsinki, Helsinki, Finland; ^2^Institute for Atmospheric and Earth System Research (INAR), University of Helsinki, Helsinki, Finland; ^3^Department of Environmental and Biological Sciences, University of Eastern Finland, Joensuu, Finland

**Keywords:** age-related effect, bayesian hierarchical modelling, whole-tree hydraulics, sap flow, stomatal conductance, leaf-sapwood area ratio

## Abstract

Age-related effects on whole-tree hydraulics are one of the key challenges to better predicting the production and growth of old-growth forests. Previous models have described the optimal state of stomatal behaviour, and field studies have implied on age/size-induced trends in tree ecophysiology related to hydraulics. On these bases, we built a Bayesian hierarchical model to link sap flow density and drivers of transpiration directly. The model included parameters with physiological meanings and accounted for variations in leaf-sapwood area ratio and the time lag between sap flow and transpiration. The model well-simulated the daily pattern of sap flow density and the variation between tree age groups. The results of parameterization show that (1) the usually higher stomatal conductance in young than old trees during mid-summer was mainly because the sap flow of young trees were more activated at low to medium light intensity, and (2) leaf-sapwood area ratio linearly decreased while time lag linearly increased with increasing tree height. Uncertainty partitioning and cross-validation, respectively, indicated a reliable and fairly robust parameter estimation. The model performance may be further improved by higher data quality and more process-based expressions of the internal dynamics of trees.

## Introduction

### Hydraulic Dynamics in Trees

Hydraulic limitation is one of the key age-related effects restraining the production and height growth of old trees (Ryan and Yoder, 1997; Koch et al., 2004; Ryan et al., 2006). Modelling and physiological theories have suggested that hydraulic conductance in trees declines with increasing transport distance, although widened conduits compensate for this loss to a limited extent (West et al., 1999; Tyree and Zimmermann, 2002; Koch et al., 2004). This age/size-related loss of water transport efficiency has been observed in a wide variety of trees and is likely to contribute significantly to the reduction of the gross primary production (GPP) of old trees and stands (e.g., McDowell et al., 2002a; Martínez-Vilalta et al., 2007; Drake et al., 2010; Olson et al., 2014). Thus, the quantification of whole-tree hydraulic dynamics is important for accurately predicting the production and growth of old-growth forests.

Simultaneously controlling the influx of carbon dioxide (CO_2_) and the efflux of water, stomata are a critical nexus between trees and the environment for both hydraulic functioning and productivity. Modelling transpiration and assimilation associated with stomatal behaviour responding to light and atmospheric water conditions has been extensively studied (e.g., Jarvis, 1976; Cowan and Farquhar, 1977; Hari et al., 1986; Leuning, 1995; Mäkelä et al., 1996; Oren et al., 1999; Hari and Mäkelä, 2003; Medlyn et al., 2011; Dewar et al., 2018). Based on physical and plant physiological analyses, these optimization or empirical models have described transpiration as a function of vapour pressure deficit (VPD) and irradiance and performed well in simulation. However, these models usually implicitly assume that evaporative demand in leaf can be satisfied by water transport through the xylem, while less attention has been paid to modelling sap flow density at similar temporal and spatial scales of the leaf processes. This modelling is difficult for two main reasons as follow.

Firstly, leaf-sapwood area ratio (*A*_L_:*A*_SW_) that differs among trees (e.g., Mencuccini and Grace, 1995; McDowell et al., 2002b; Domec et al., 2012) introduces variability into the conversion between transpiration rate (water transpired per *leaf* area) and sap flow density (per *sapwood* area). Secondly, the time lag between evaporative demand and sap flow mainly due to transport time and storage processes must be quantitatively described for better model performance (Perämäki et al., 2001; Sevanto et al., 2002). Overcoming these challenges should help bridge spatial and temporal scales of tree ecophysiology because (1) whole-tree hydraulic dynamics, from sap flow to transpiration, can be more accurately predicted and (2) the daily patterns of these dynamics can be described more precisely in fine temporal units (e.g., minute).

Directly linking the drivers of transpiration and sap flow should also broaden the use of sap flow measurement to research on whole-tree scale. By the current methods, measurement of sap flow is easier technically and lower in costs than that of transpiration. The decades-long practice of sap flow measurements by different means has developed accurate and economical methods and adequate discussion on uncertainties (e.g., Cermák et al., 1973, 2004; Granier, 1987; Köstner et al., 1998; Oren et al., 1998; Clearwater et al., 1999; Burgess et al., 2001; Oishi et al., 2008; Steppe et al., 2010; Cermák and Nadezhdina, 2011; Hölttä et al., 2015; Berdanier et al., 2016). Should the two major challenges be overcome, sap flow measurements provide more insights into whole-tree hydraulic dynamics as well as tree age/size-related changes in stomatal behaviour and tree structure, whereas the scale-up of transpiration measurements are usually limited by the considerable heterogeneity among sample leaves or shoots and can hardly imply on ageing effects at whole-tree level. In consideration of these challenges and potentials, we developed a Bayesian hierarchical model to link environmental drivers and the sap flow density in Scots pine (*Pinus sylvestris* L.) trees, a dominant species of the Eurasian boreal forests, with the variabilities of *A*_L_:*A*_SW_ and time lag being addressed. This Bayesian inference constitutes an inverse method for estimating stomatal conductance of the tree as a “big leaf” and quantifies its sensitivity to the driving forces, which cannot be measured by sampling individual shoots' transpiration rates. Using this model on young and old trees, we also intended to quantify the tree age/size-related effects on stomatal behaviour and structural features (*A*_L_:*A*_SW_ and internal water storage) and to provide insights into the physiological reasons behind the differences in their stomatal conductance.

### Bayesian Hierarchical Modelling and Hypotheses

The Bayesian hierarchical model in this study comprises three levels, namely, the process, data, and parameter models (Dietze, 2017). The process model defines at leaf level the optimum of net carbon gain constrained by water loss regulated by stomatal behaviour. The modelled stomatal conductance is compared with measured sap flow density at tree base in the data model to calculate the error, and therein *A*_L_:*A*_SW_ and time lag are introduced as parameters for bridging the whole-tree hydraulic dynamics. The parameter model describes the variabilities of *A*_L_:*A*_SW_ by a probability density function, of which the parameters are suggested by literature. Focussing on the age-related ecophysiological effects represented by the process and data models' parameters, we proposed and tested the following hypotheses on time lag, *A*_L_:*A*_SW_, and stomatal conductance.

Concerning the time lag between sap flow and transpiration dynamics, observations and models have suggested that whole-tree hydraulic resistance increases with tree height (Mencuccini and Grace, 1996a; Niklas and Spatz, 2004). Additionally, water storage should increase with tree size (Hölttä et al., 2009; Scholz et al., 2011). The larger resistance (*R*) and capacitance (*C*) in older/taller trees suggest that their hydraulic system requires a longer time to reach a steady state after fluctuation in evaporative demand, featured with a larger time constant (τ = *RC*; Jones, 2014).

Tree age/height-related declines in *A*_L_:*A*_SW_ (*A*_SW_ measured at breast height) and stomatal conductance in conifers have been considered as characteristics of conservative water use against increased evaporative demand and risk of embolism in the xylem (e.g., Whitehead et al., 1984; McDowell et al., 2002b, 2008; Ewers et al., 2005; Magnani et al., 2008; Steppe et al., 2011). In Scots pine, declining *A*_L_:*A*_SW_ with tree height has been found in various environmental conditions (e.g., Mencuccini and Grace, 1995; Vanninen et al., 1996; McDowell et al., 2002b; Martínez-Vilalta et al., 2007; Poyatos et al., 2007). Also, declining stomatal conductance with tree age/height in Scots pine has been observed in previous studies (e.g., Niinemets, 2002; Martínez-Vilalta et al., 2009). Hence, we hypothesized that the parameterization of the model manifests higher *A*_L_:*A*_SW_ and stomatal conductance and lower time lag in young Scots pine trees.

## Materials and Methods

### Study Sites and Sample Trees

The study sites were located by the Hyytiälä Forest Station (61.8°N, 24.3°E), University of Helsinki, and Station II for Measuring Forest Ecosystem-Atmosphere Relations (SMEAR II; Hari and Kulmala, 2005) in southern Finland. The sap flow data were collected in July 2018. Scots pine is the dominant species in the area, and other common tree or tall shrub species include Norway spruce (*Picea abies* (L.) H. Karst.), silver and downy birches (*Betula pendula* Roth and *B. pubescens* Ehrh.), goat willow (*Salix caprea* L.), the Eurasian aspen (*Populus tremula* L.) and rowan (*Sorbus aucuparia* L.). The site of young Scots pine was a sub-xeric (site type VT; Lehto, 1964) monospecific stand clear cut for the last time at *c*. 1960, while that of old pine is a mesic grove-like (site type MT to OMT; Lehto, 1964) mixed stand with Norway spruce that had not been thinned since the 1920's. Sap flow density was measured in July 2018 on six sample trees in each of the sites (Table 1), but the data of one young sample tree were not used due to noises. The measurement was conducted in two rounds with trees O1 and Y1—Y5 in the first (day of year [DOY] 190−199) and O2−6 in the second (DOY 199−208). Tree ages were determined by coring at breast height. During 2009–2018, the summer (June and July) mean air temperatures at the heights of the young and the old canopies were 15.3 °C and 15.0 °C, respectively, and the summer mean cumulative precipitation was 190.9 mm (https://avaa.tdata.fi/web/smart/smear).

**Table 1:** Information on sample trees.

**Young**				**Old**			
**No**.	**Age**	***H***	**DBH**	**No**.	**Age**	***H***	**DBH**
	**(yr)**	**(m)**	**(cm)**		**(yr)**	**(m)**	**(cm)**
Y1	52	18.1	20.5	O1	161	33.3	48.6
Y2	52	20.9	23.9	O2	177	31.0	43.0
Y3	50	16.0	18.5	O3	133	36.8	47.3
Y4	50	17.1	17.3	O4	132	33.7	49.2
Y5	52	20.4	22.3	O5	137	35.9	46.6
				O6	154	34.8	51.5
Mean	51	18.5	20.9	Mean	149	34.3	47.7
(SD)	(1.0)	(1.7)	(2.4)	(SD)	(16.5)	(1.9)	(2.6)

*H, tree height; DBH, diameter at breast height; SD, standard deviation*.

*H was measured by airborne LiDAR [as described by Korpela et al. (2010)] in 2016. Age was counted at breast height in 2018*.

### Measurement of Sap Flow Density

The thermal dissipation method (Granier, 1987) was employed to collect the raw data of sap flow density. Four pairs of probes were mounted to each sample tree, each pair comprising a heated probe (HP; higher on the trunk) and a reference probe (RP; lower) with a *c*. 10-cm interval. Each HP and RP contains a Type T (copper-constantan) thermocouple. The probes were located on northwest, southeast (HP at *c*. 130 cm from ground), southwest and northeast (HP at *c*. 115 cm from ground), orientated by compass. The difference in mounting heights was to minimize thermal interference among the probes. Brass tubes filled with thermal grease in a diameter barely larger than the probes were applied to all of HP and RP to protect them and to facilitate thermal conductance. An increment core was obtained by each HP after the measurement to define the corresponding sapwood thickness. All pairs of probes were covered by aluminium foil to mediate the effects of direct sunlight.

A constant power of 0.2 W (Lu et al., 2004) was supplied to each HP during the measurement. The voltage difference between HP and RP (Δ*U*, mV) was read by data logger (Type DL2e by Delta-T Devices Ltd, UK) every minute, and the 10-min mean was recorded. Following Oishi et al. (2008), the time point(s) of baseline voltage (ΔU^*^, mV) of each pair of probes was defined as below:

1. The 2-hour average VPD (Equation 2) is not larger than 50 Pa; AND
2. The 2-hour standard deviation of Δ*U* is not larger than 5‰ of the mean of Δ*U* over the same period that fulfils (1).

The raw sap flow density (*J*, m s^−1^) was converted from voltage by Granier (1987); Lu et al. (2004)

(1) $$\begin{array}{r} J=118.99\times 10^{-6}{\left(\frac{\Delta U^{*}-\Delta U}{\Delta U}\right)}^{1.231} \end{array}$$

The whole-tree sap flow density (*J*_*m*_ in mol m^−2^ SW s^−1^, where *m* denotes the ordinal number of the tree as in Table 1) was calculated from *J* with the locations of probes and the respective sapwood thickness considered (see Supplementary Material for details).

### Data of Environmental Factors

The data of air temperature (*T*, °C) and relative humidity (*h*_*r*_, %) at heights of 16.8 and 33.6 m were collected from the SMEAR system (https://avaa.tdata.fi/web/smart/smear). These heights were close to those of the young and old canopies (Table 1), respectively, and referred to as the lower and the higher heights hereafter. The data of over-canopy photosynthetic photon flux density (PPFD, mol m^−2^ s^−1^; over the waveband 400–700 nm, at height = 35 m) and atmospheric CO_2_ concentration were also collected from the SMEAR system. VPD was calculated from *T* and *h*_*r*_ by Jones (2014)

(2) $$\begin{array}{r} VPD=\left(1-\frac{h_{r}}{100}\right)\left(1.0007+3.46\times 10^{-8}P_{0}\right)\\ \;\left[611.21\;\exp\left(\frac{18.678-\frac{T}{234.5}}{257.14+T}\right)\right] \end{array}$$

where the standard atmospheric pressure *P*_0_ = 101325 Pa. It was converted to the unit of mol m^−3^ by the ideal gas law to be in accordance with the process model (Equations 4, 5):

(3) $$\begin{array}{r} D=\frac{VPD}{R\left(T+273.15\right)} \end{array}$$

where the ideal gas constant *R* = 8.3145 J K^−1^ mol^−1^.

**Figure 1:**
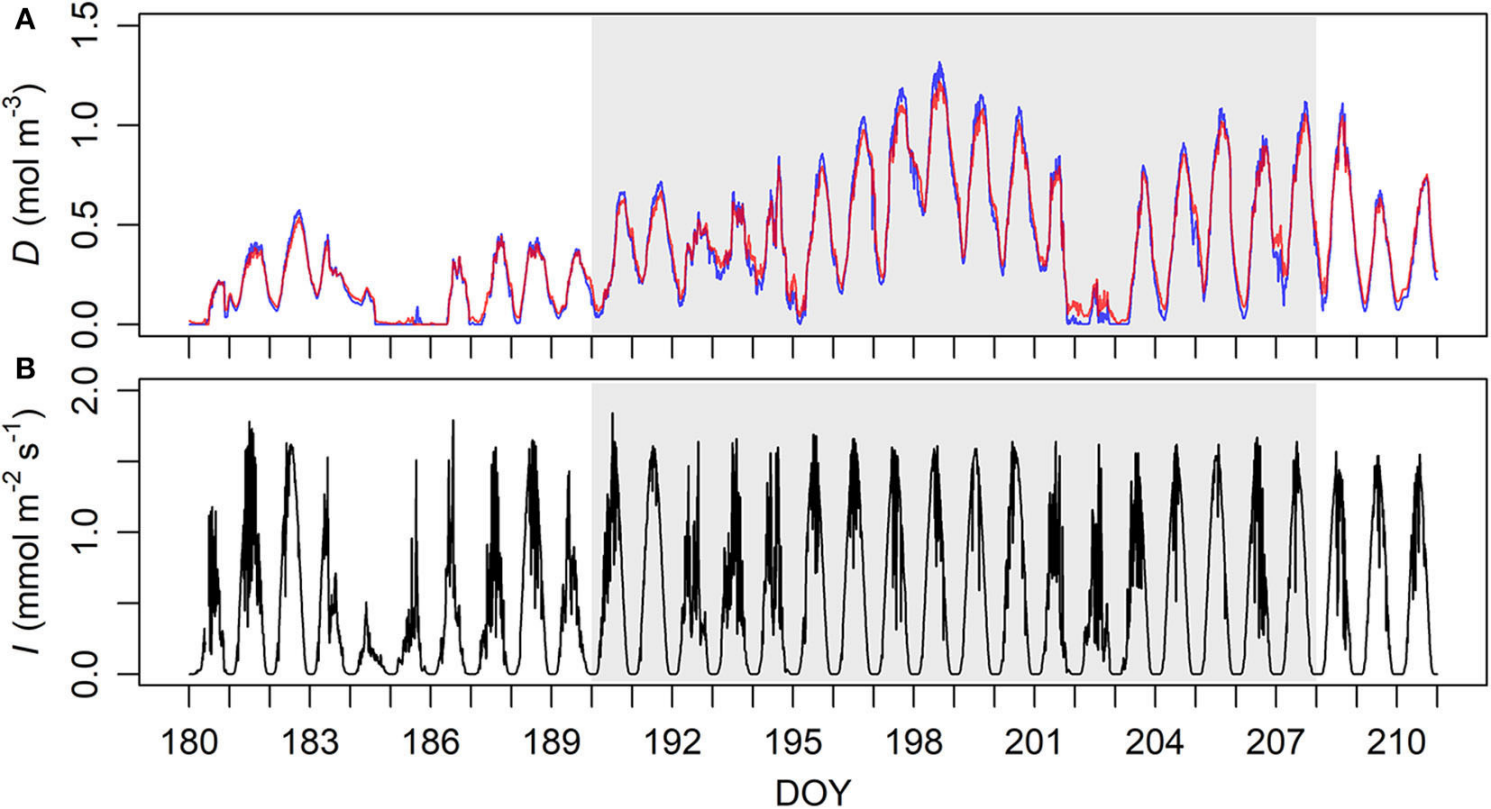
Ten-minute means of (**A**, top) vapour deficit (*D*) and (**B**, bottom) photosynthetic photon flux density (*I*) at the study sites during late June to late July 2018. **(A)** *D* near the canopy heights of the old (33.6 m, red) and the young (16.8 m, blue) sample trees' sites. **(B)** Measurement height 35 m. DOY, day of year. The period of sap flow measurement is demarcated by grey shades.

The original time interval of the data of VPD and PPFD was 1 min, and their 10-min means were calculated for the synchrony with the sap flow data. The data with *D* = 0 were removed as they are not allowed arithmetically in the current process model (Equation 4). The numbers of valid data points were 6,510 and 4,862 of the old and the young sample trees, respectively. *D* was very similar at the higher and lower heights, and positively correlated with PPFD at moderate level at both heights (Figure 1, Supplementary Table 1).

### The Hierarchical Model

In the process model, the optimal steady state of gas exchange at stomata is defined as the maximum carbon gain constrained by water loss (Hari et al., 1986; Berninger and Hari, 1993). As such, the evaporation rate of water (*E*, mol m^−2^ s^−1^) at the stomatal aperture is proportional to the conductance to water vapour (*g*, m s^−1^) and *D* (mol m^−3^) (Hari et al., 1986; Jarvis and McNaughton, 1986; Jones, 2014). By Lagrangian optimization and introducing an irradiance (*I*, i.e. PPFD) response curve (Mäkelä et al., 1996, 2004; Hari and Mäkelä, 2003), the stomatal conductance at the optimal steady state can be solved, and thus transpiration rate can be modelled as

(4) $$\begin{array}{r} E^{\left(\text{M}\right)}\left(t\right)=g\left(t\right)D\left(t\right)=\left[1.6\left(\sqrt{\frac{C_{\text{a}}}{1.6\;\lambda D\left(t\right)}}-1\right)\frac{\iota \gamma I\left(t\right)}{\iota I\left(t\right)+\gamma }\right]D\left(t\right) \end{array}$$

where 1.6 is the ratio of the diffusion rates of water vapour to CO_2_ in air (e.g., Lambers et al., 2008), *C*_a_ the atmospheric concentration of CO_2_ (mol m^−3^), λ marginal carbon gain per water loss (mol CO_2_ mol^−1^ H_2_O), and ι (m^3^ mol^−1^) and γ (m s^−1^) the initial slope and the saturation level, respectively, of the irradiance response curve (IRC). *C*_a_ was evaluated by the mean of the records at SMEAR II over the period of fieldwork (402.73 ± 8.11 ppm or (1.701 ± 0.034) × 10^−2^ mol m^−3^).

Two assumptions were made when parameterizing *A*_L_:*A*_SW_ (noted ρ in the model) and the time lag between *J* and *E*: Through the measurement period, (1) the summed mass of the sap flow at tree base was equal to that of transpiration, i.e. *A*_*SW*_∫*J*(*t*)*dt* = *A*_*L*_∫*E*(*t*)*dt* [but see Hölttä et al. (2006) on the minor amount of translocated water via phloem with assimilates]; and (2) the time lag (χ) was constant in each sample tree. The first assumption allowed a tree-specific (all-sided) leaf to sapwood area ratio (ρ_*m*_) being a multiplier to Equation 4 to model the sap flow density (*J*^(M)^) at a time (*t*) in one sample tree (*m*), i.e.

(5) $$\begin{array}{r} J_{m,t}^{\left(M\right)}=E_{m,t}^{\left(M\right)}\times \rho_{m}=1.6\;\rho_{m}D_{t}\left(\sqrt{\frac{C_{\text{a}}}{1.6\;\lambda_{\text{AG}}D_{t}}}-1\right)\frac{\iota_{\text{AG}}\gamma_{\text{AG}}I_{t}}{\iota_{\text{AG}}I_{t}+\gamma_{\text{AG}}} \end{array}$$

where AG denotes age group (young or old), and *C*_a_, λ, ι and γ have the same meanings and units as in Equation 4.

In the data model, the tree-specific time lag (χ_*m*_) between the modelled *J*^(M)^ and observed *J*^(O)^ was represented by linear interpolation of two nearest time points. Hence, the error (ε) between *J*^(M)^ and *J*^(O)^ at each time point with χ_*m*_ considered can be expressed as

(6) $$\begin{array}{r} \varepsilon_{m,t}=\left[\frac{\left(\chi_{m}-t_{0}\right)J_{m,t+t_{0}}^{\left(\text{O}\right)}+\left(t_{1}-\chi_{m}\right)J_{m,t+t_{1}}^{\left(\text{O}\right)}}{10}\right]-J_{m,t}^{\left(\text{M}\right)} \end{array}$$

where *t*_0_ and *t*_1_ are the earlier and later measurement time points (*t*_1_-*t*_0_ = 10 min, i.e., the time interval between two adjacent measurement points) in the observations, respectively, nearest to χ_*m*_. The Laplace distribution was chosen to be the probability density function (PDF) of ε, of which the rate parameter differs between young and old trees. The openness of the young tree site might cause heteroscedasticity with *J*^(M)^ as direct sunlight reached the bases of trunks, although aluminium foils were applied to mediate this effect. Correspondingly, the rate parameter of young trees' ε was in correlation with *J*^(M)^ (and thus air temperature) with a positive initial slope (*a*) and an asymptote (*b*). In contrast, the canopy was closed in the old tree site, and the measurement error was considered random and constant over the measurement period. Hence,

(7) $$\begin{array}{l} p\left(\varepsilon \right)=\prod_{m_{\text{Y}}\;=\;1}^{5}\prod_{t\;=\;1}^{N_{m_{\text{Y}}}}\mathrm{Laplace}\left(0,c_{\text{Y}}+\frac{a_{m_{\text{Y}}}b_{m_{\text{Y}}}J_{m_{\text{Y}},t}^{\left(\text{M}\right)}}{a_{m_{\text{Y}}}J_{m_{\text{Y}},t}^{\left(\text{M}\right)}+b_{m_{\text{Y}}}}\right)\\ \;\prod_{m_{\text{O}}=1}^{6}\prod_{t\;=\;1}^{N_{m_{\text{O}}}}\mathrm{Laplace}\left(0,c_{\text{O}}\right)\\ \;={\prod_{m_{\text{Y}}\;=\;1}^{5}\prod_{t\;=\;1}^{N_{m_{\text{Y}}}}\frac{1}{2}\left(c_{\text{Y}}+\frac{a_{m_{\text{Y}}}b_{m_{\text{Y}}}J_{m_{\text{Y}},t}^{\left(\text{M}\right)}}{a_{m_{\text{Y}}}J_{m_{\text{Y}},t}^{\left(\text{M}\right)}+b_{m_{\text{Y}}}}\right)}^{-1}\\ \;\exp\left(-\frac{\left|\varepsilon_{m_{\text{Y}},t}\right|}{\frac{a_{m_{\text{Y}}}b_{m_{\text{Y}}}J_{m_{\text{Y}},t}^{\left(\text{M}\right)}}{a_{m_{\text{Y}}}J_{m_{\text{Y}},t}^{\left(\text{M}\right)}+b_{m_{\text{Y}}}}+c_{\text{Y}}}\right)\prod_{m_{\text{O}}\;=\;1}^{6}\prod_{t\;=\;1}^{N_{m_{\text{O}}}}\frac{1}{2c_{\text{O}}}\\ \;\exp\left(-\frac{\left|\varepsilon_{m_{\text{O}},t}\right|}{c_{\text{O}}}\right) \end{array}$$

where *N*_*m*_Y__ and *N*_*m*_*O*__ denote the numbers of data points of trees *m*_Y_ (young) and *m*_O_ (old), respectively, and *a, b* and *c* (all > 0) are parameters of the rates of the Laplace distributions; the upper bounds of the outer multiplication (5 and 6) refer to the numbers of young and old trees, respectively (Table 1).

The parameter model describes the variabilities of *A*_L_:*A*_SW_ (noted ρ). Regarding the decline of this ratio with increasing tree height (*H*) in Scots pine (McDowell et al., 2002b), the young and the old trees were separated into two distributions of the same form, that is, a heavy-tailed Gaussian distribution following Sivia and Skilling (2006). The heavy-tailed distribution was chosen because at least one old sample tree likely presented an outlying estimated ρ. Hence, the joint posterior distribution of the parameters given the observations is

(8) $$\begin{array}{l} p\left(\theta ,\mu ,\sigma |J^{\left(\text{O}\right)}\right)\propto p\left(\varepsilon \right)\times \;\\ \prod_{m_{\text{Y}}=1}^{5}\frac{1}{\sigma_{\text{Y}}\sqrt{2\pi }}\left[\frac{1-\exp\left(-\frac{{\left(\frac{\left(\rho_{m_{\text{Y}}}-\mu_{\text{Y}}\right)}{\sigma_{\text{Y}}}\right)}^{2}}{2}\right)}{{\left(\frac{\left(\rho_{m_{\text{Y}}}-\mu_{\text{Y}}\right)}{\sigma_{\text{Y}}}\right)}^{2}}\right]\\ \prod_{m_{\text{O}}=1}^{6}\frac{1}{\sigma_{\text{O}}\sqrt{2\pi }}\left[\frac{1-\exp\left(-\frac{{\left(\frac{\left(\rho_{m_{\text{O}}}-\mu_{\text{O}}\right)}{\sigma_{\text{O}}}\right)}^{2}}{2}\right)}{{\left(\frac{\left(\rho_{m_{\text{O}}}-\mu_{\text{O}}\right)}{\sigma_{\text{O}}}\right)}^{2}}\right]\; \end{array}$$

where **ϑ** = {**λ**, **ι**, **γ**, **ρ**, **χ**} are the parameters of the process and data models (Equations 5, 6), and **μ** = {μ_Y_, μ_O_} and **σ** = {σ_Y_, σ_O_} are the mean and the variance parameters of the heavy-tailed distribution of ρ of the young and old sample trees, respectively; *p*(**ε**) is given in Equation 7. All the parameters but ρ were assumed independently distributed and to follow the uniform distribution. Thus, their probabilities were omitted from the right-hand side of Equation 8. By this point, the hierarchical model was complete, and the parameters representing whole-tree hydraulic dynamics were nested into the same framework.

### Model Parameterization, Parametric Uncertainty and Cross-Validation

The parameters were estimated by Markov chain Monte Carlo (MCMC) algorithm DREAM_(ZS)_ (Vrugt et al., 2009). The estimation was performed by R 3.5.3 (R Core Team, 2019) with package “BayesianTools” (Hartig et al., 2019). The total number of parameters was 44 (see Table 2 for the meanings, units and initial ranges of λ, ι, γ, ρ and χ, and Supplementary Tables 2B, 3A for a full list of all parameters). The standard of convergence was $\hat{R}$ < 1.1, and only the second halves of the random walk chains were used for the convergence test and all the subsequent analyses (Gelman et al., 2014).

**Table 2:** Information on the hypothesis-related parameters of the model.

	**Spec**.	**Meaning (unit)**	**Prior range (min., max.)**	**References**
λ	AG	Marginal carbon gain per water cost (mol CO_2_ mol^−1^ H_2_O)	0.5 × 10^−3^, 6.0 × 10^−3^	1
ι	AG	Initial slope of PPFD response curve (m^3^ mol^−1^)	0.1, 1.2	1
γ	AG	Saturation level (asymptote) of PPFD response curve (m s^−1^)	1.6 × 10^−3^, 5.0 × 10^−3^	2–4
ρ	tree	(All-sided) Leaf to sapwood area ratio (m^2^ leaf m^−2^ SW)	Y: 3,000, 6,500 O: 2,500, 6,000	5, 6
χ	tree	Time lag between modelled transpiration and measured sap flow density (minute)	1, 18	7

*Spec., specificity of parameter estimates, where AG and tree denote age group- and tree-specific, respectively*.

*Y, young trees, and O, old trees, if initial ranges differed*.

*Ref., references of the initial ranges: (1) Hari and Mäkelä (2003), (2) Mäkelä et al. (2004), (3) Markkanen et al. (2001), (4) Kolari et al. (2014), (5) Vanninen et al. (1996), (6) McDowell et al. (2002b), and (7) Phillips et al. (1997). The maximum of the initial range of γ was tuned during this study because the value suggested by Ref. 2−4 truncated its posterior distribution significantly*.

The predictive uncertainty of the model was generated by Equation 7 and partitioned into parametric and measurement uncertainties. To determine the parametric uncertainty, 3,000 parameter vectors were randomly sampled from the joint posterior distribution (Equation 8) using MCMC. The measurement uncertainty was the difference between the predictive and parametric uncertainties, reflecting the distributions of sap flow density that could probably occur. These distributions were truncated at zero as negative values of sap flow density lack physical meaning.

An eight-fold cross-validation was performed. Within Days 1−8 of all trees, 1 day of data were removed in turn, and the remaining data were used to parameterize the model. The eight parameter estimations and their performances in simulating the removed day were compared with those by using the full data set for testing the robustness of parameterization. Considering the correlation between ι and γ suggested by Equation 4, the cross-validation was conducted twice with either ι_Y_ and ι_*O*_ or γ_Y_ and γ_*O*_ fixed to their maxima a posteriori (MAP) estimates with the full data.

### Sensitivity of Stomatal Conductance to Environmental Factors

To clarify the cause of difference between stomatal conductance of young and old trees, we used the partial derivatives (∂*g*/∂*D* and ∂*g*/∂*I*) and a relative sensitivity coefficient to measure the sensitivities of *g* to *D* and *I*. The relative sensitivities $C_{D}^{g}$ and $C_{I}^{g}$ are defined as (Kacser and Burns, 1973; Kacser and Fell, 1995)

(9) $$\begin{array}{r} C_{D}^{g}=\frac{\partial g}{\partial D}/\frac{g}{D}=\frac{1}{2\sqrt{\frac{1.6\;\lambda D}{C_{a}}}-2} \end{array}$$

(10) $$\begin{array}{r} C_{I}^{g}=\frac{\partial g}{\partial I}/\frac{g}{I}=\frac{\gamma }{\iota I+\gamma } \end{array}$$

which were calculated from the process model (Equation 4) with the MAP estimates of λ, ι, and γ.

### Statistical Analyses

To assess the performance of the model, the tree-specific and all-data root-mean-square error (RMSE) was calculated as

(11) $$\begin{array}{r} \text{RMSE}=\sqrt{\frac{\sum_{t\;=\;1}^{N}\varepsilon_{t}^{2}}{N}} \end{array}$$

where *N* is the total number of data points of concern, and ε the same as in Equations 6, 7 calculated by the MAP estimates of the parameter vectors. RMSE was also calculated in the eight-fold cross-validation, in which each fold was treated as an independent model parameterization. The normalized RMSE (RMSE%) was defined as

(12) $$\begin{array}{r} \text{RMSE\%}=\frac{RMSE}{\bar{J^{\left(\text{O}\right)}}}\times 100 \end{array}$$

where $\bar{J^{\left(\text{O}\right)}}$ is the mean of *J*^(O)^ of the corresponding tree or the entire data.

Linear regression was performed on *J*^(M)^ to *J*^(O)^ as well as the MAP estimates of tree-specific ρ or χ to tree height (*H*) (by R 3.5.3).

## Results

### Model Performance and Robustness

**Figure 2:**
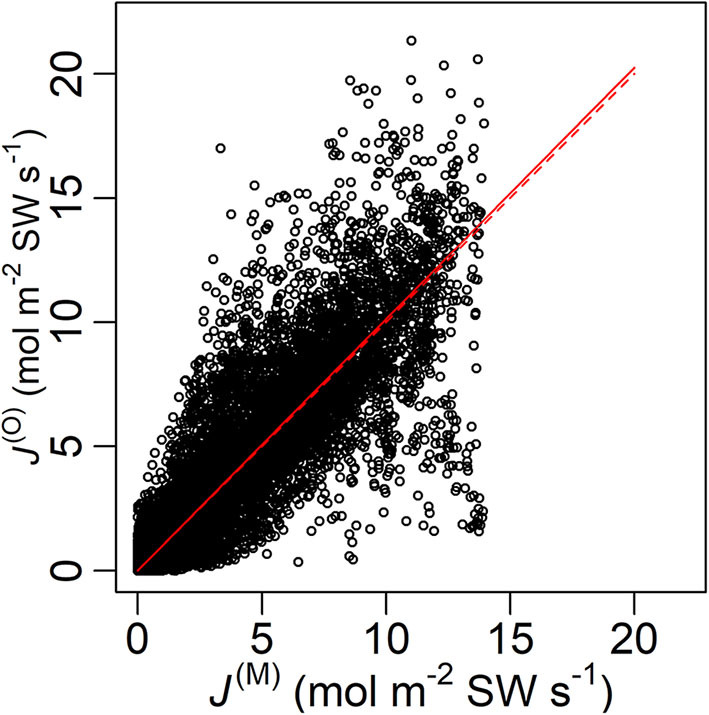
Modelled (*J*^(M)^) and measured (*J*^(O)^) sap flow density of all data points with a fitted line (solid) and the line *y* = *x* (dashed). Root-mean-square error (RMSE) = 1.85 mol m^−2^ SW s^−1^, fitted slope = 1.01, *R*^2^ = 0.755, *P* < 0.0001. SW, sapwood.

**Figure 3:**
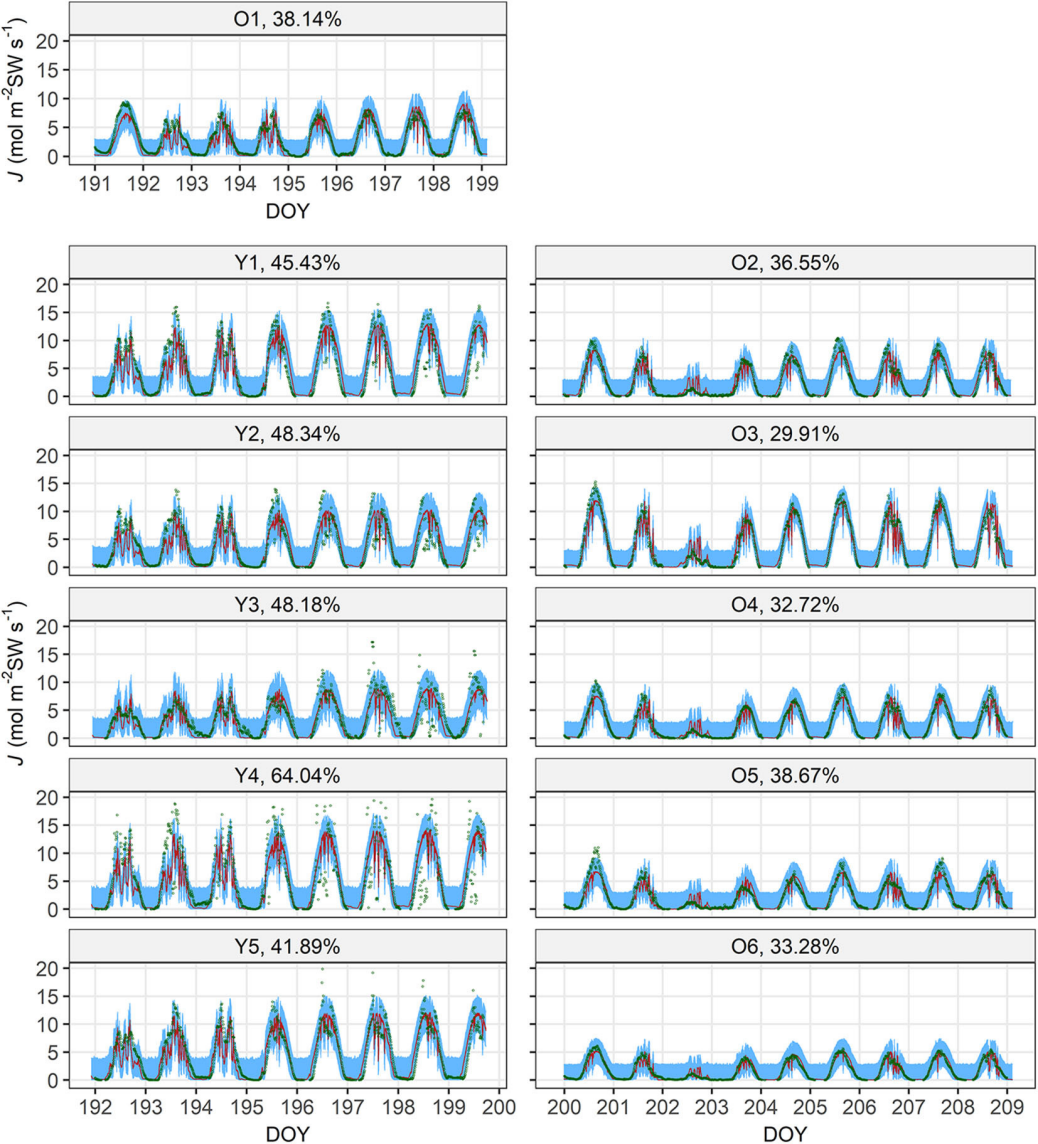
Measured sap flow density (*J*^(O)^, dark green circle) and 95% intervals of parametric (λ, ι, γ, ρ and χ; red shade) and measurement (blue shade) uncertainties of modelled sap flow density (*J*^(M)^) of the sample trees. Each panel is titled with the tree's number (Table 1) and normalized root-mean-square error (RMSE%) by the mean of respective *J*^(O)^. For visual clarity, the *J*^(M)^ values with the maxima a posteriori (MAP) estimates of the parameter vectors are not shown because the parametric uncertainty is very small. DOY, day of year (marked at 00:00 of the day).

The model performed well on the full data, judged by the good linear fit between *J*^(M)^ and *J*^(O)^ (*R*^2^ = 0.758) with a slope = 1.01 (Figure 2). The tree-specific performance, however, differed between age groups, with RMSE% < 40% for all the old sample trees but RMSE% > 40% for the young ones (Figure 3). In all the sample trees, the measurement error was noticeably larger than the parametric error.

Most estimates of the parameter vector **ϑ** were robust in the cross-validation with, however, several exceptions including γ_Y_ when ι was fixed and χ_*Y*4_ and χ_*Y*5_ when either ι or γ was fixed (Supplementary Figures 1A,B). The model performance was robust, judged by the fairly stable RMSE throughout the cross-validation.

### Parameter Estimates of Stomatal Conductance

**Figure 4:**
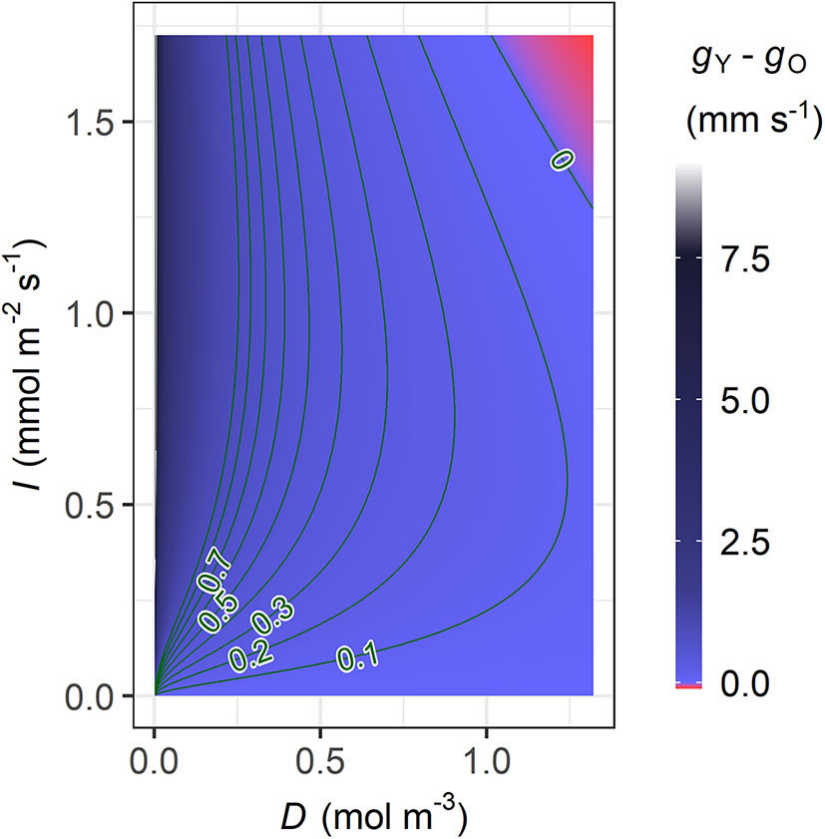
Contour plot of the difference in stomatal conductance between the young and old sample trees (*g*_Y_−*g*_*O*_, mm s^−1^) modelled with the maxima a posteriori (MAP) estimates of λ, ι, and γ (Table 3A), in relation to vapour deficit (*D*) and photosynthetic photon flux density (*I*). The colour legend of *g*_Y_−*g*_*O*_ (right) ranges up to the *g* value that allowed the maximum sap flow density during the measurement period, and the higher values are coloured in grey. The lowest value in the red area is −9.54 × 10^−2^ mm s^−1^.

**Figure 5:**
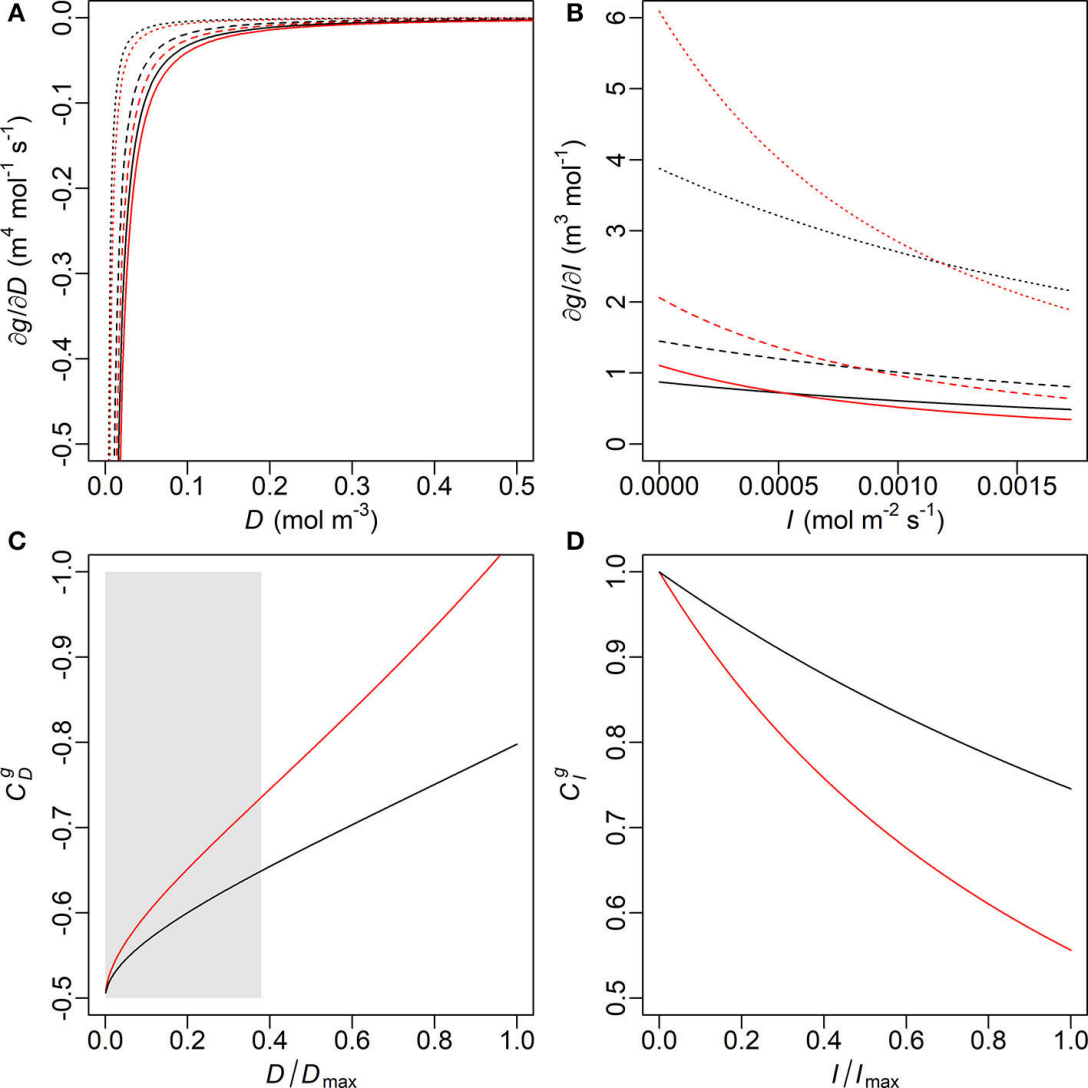
Partial derivatives of stomatal conductance (*g*) with respect to (**A**, top left) vapour deficit (*D*) and (**B**, top right) photosynthetic photon flux density (*I*) and the relative sensitivity coefficients (**C**, bottom left) ${\text{C}}_{\text{D}}^{\text{g}}$ (with reversed *y*-axis) and (**D**, bottom right) ${\text{C}}_{\text{I}}^{\text{g}}$, calculated with the maxima a posteriori (MAP) estimates of λ, ι and γ of the young (red) and old (black) sample trees (Table 3A). Solid, dashed and dotted lines designate the conditions that the other variable [*I* in **(A)**, *D* in **(B)**] is held constant at its level of 100, 50, and 10% maximum of the measurement period, respectively. Such maxima values are noted as *D*_max_ and *I*_max_ in **(C)** and **(D)**, respectively. Only limited ranges of *D* and ∂*g*/∂*D* are displayed in **(A)** for visual clarity, which correspond to the grey box in **(C)**.

In the young trees, the MAP estimate of the initial slope of IRC (ι) is higher but the marginal carbon gain per water loss (λ) is also higher, while the saturation of IRC (γ) is insignificantly higher in the old trees (Table 3A and Supplementary Table 3B). Nevertheless, modelled stomatal conductance (*g*) is usually higher in the young than the old sample trees unless both *D* and *I* are very high (Figure 4). In the latter case, however, the difference (|*g*_*O*_−*g*_Y_|) always remains lower than 0.1 mm s^−1^. In original units, the sensitivity of *g* to *D* under the same condition of *I* is similar in both age groups and stabilized at a constant level close to zero since low *D*, although the young trees are slightly more sensitive at very low levels of *D* (Figure 5A). Given the same *D*, *g*_Y_ is much more sensitive to *I* than *g*_*O*_ at low to medium *I* but less sensitive to *I* when *I* increases to high levels, and both ∂*g*_Y_/∂*I* and ∂*g*_*O*_/∂*I* responded to changes in *I* throughout the measured range (Figure 5B). The relative sensitivities of *g* to *D* and *I* ($C_{D}^{g}$ and $C_{I}^{g}$, respectively) show that when *D* and *I* were low both *g*_Y_ and *g*_*O*_ were governed by *I* with $C_{I}^{g}$ double of $C_{D}^{g}$ (Figures 5C,D). All trees' $C_{D}^{g}$ decreased with increasing *D* and *I*, but that of the young trees decreased faster. Correspondingly, the decline in $C_{I}^{g}$ of the young trees was also faster than that of the old.

**Table 3:** Maxima a posteriori (MAP) estimates of **(A)** λ, ι, γ and **(B)** ρ and χ.

**(A)**			
**Age group**	**λ**	**ι**	**γ**
Young	2.182 × 10^−3^	1.200	2.594 × 10^−3^
Old	1.124 × 10^−3^	0.520	2.625 × 10^−3^
**(B)**			
**Tree**	***ρ***		***χ***
Y1	6,003		104.5
Y2	4,774		105.1
Y3	4,100		104.2
Y4	6,493		74.3
Y5	5,551		70.4
O1	4,172		165.8
O2	4,084		122.7
O3	5,926		164.7
O4	3,728		174.7
O5	3,317		165.5
O6	2,564		168.5

*See Table 2 for units. See Supplementary Table 3B for the 95% credible intervals and the results of the other parameters*.

### Leaf-Sapwood Area Ratio, Time Lag and Tree Height

**Figure 6:**
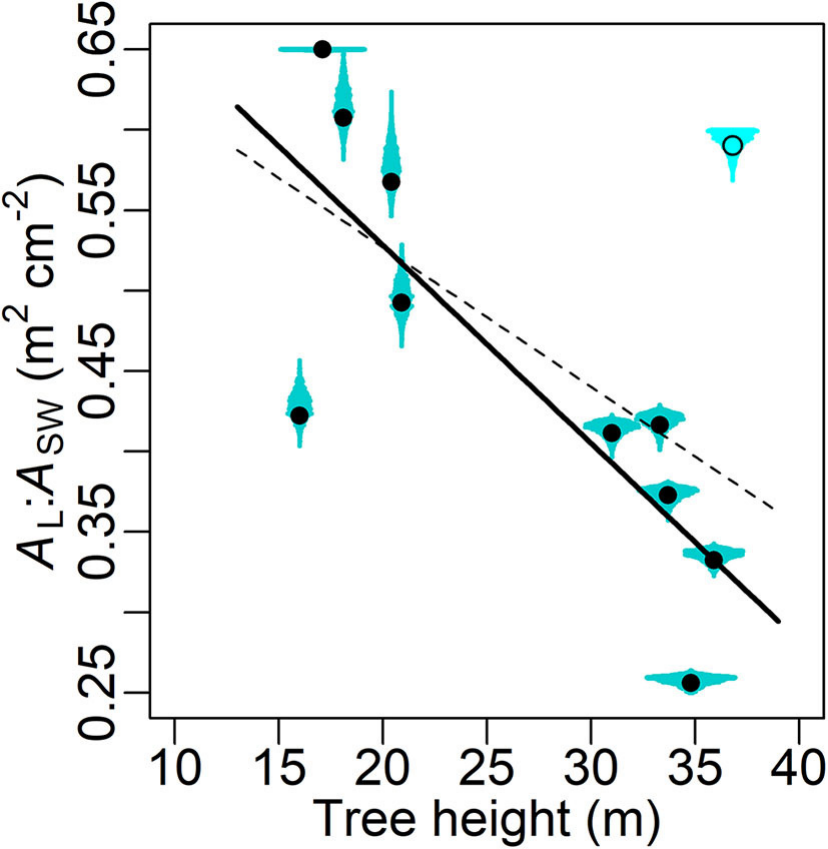
Maxima a posteriori (MAP) estimates (circle) of leaf-sapwood area ratio (*A*_L_:*A*_SW_ in m^2^ cm^−2^, i.e. ρ × 10^−4^; Table 3B) with posterior distribution (cyan shade) in relation to tree height. The dashed and solid lines, respectively, are linear fittings with and without an outlier (Cook's distance = 0.69 when used in the linear regression), which is marked by the empty circle with the shade in lighter colour. The fittings' forms *y* = −0.0087 *x* + 0.700 and *y* = −0.0123 *x* + 0.774, *R*^2^ = 0.339 and 0.657, *P* = 0.06 and 0.004, respectively.

**Figure 7:**
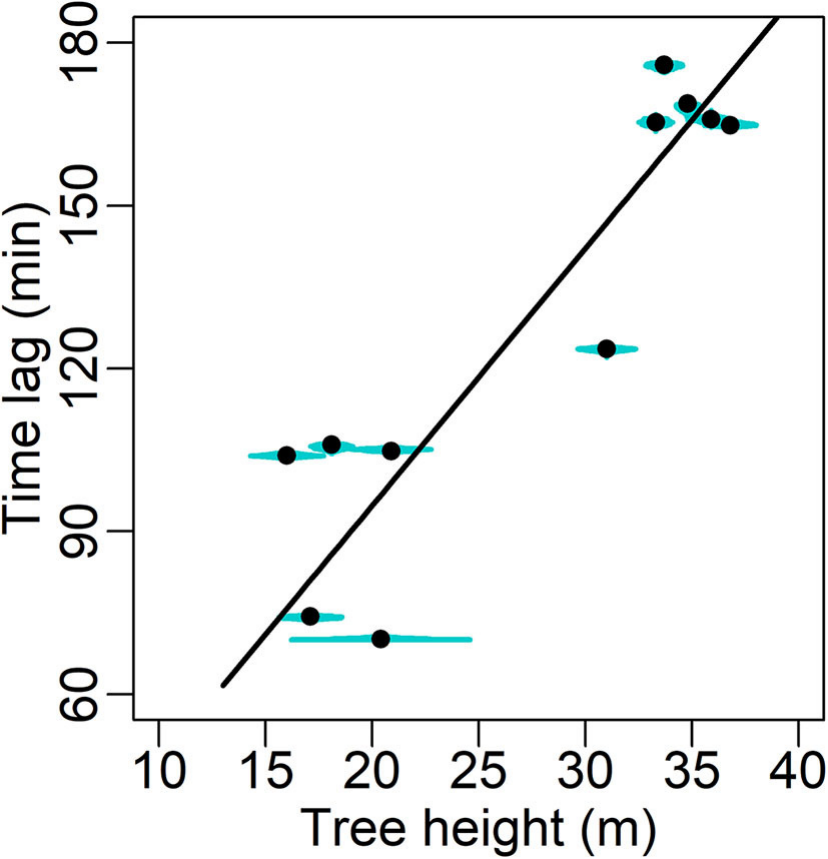
Maxima a posteriori (MAP) estimates (solid circle) of the time lag between sap flow and transpiration (min; Table 3B) with posterior distribution (cyan shade) in relation to tree height. The fitting's form *y* = 4.74 *x, R*^2^ = 0.814, *P* < 0.0001.

Excluding one outlier (Cook's distance = 0.69 when used in linear regression), a significant decline in the MAP estimates of ρ (Table 3B) with increasing tree height (*H*) is shown (slope = −0.0123 m^2^ cm^−2^ m^−1^, *R*^2^ = 0.657, *P* = 0.004; Figure 6). This outlier (O3 in Table 1) was also the main reason why a heavy-tailed distribution was selected in the parameter model (Equation 8). Using standardized ρ and *H* by the respective maxima (McDowell et al., 2002b), the slope [i.e., *d*(ρ/ρ_max_)/*d*(*H*/*H*_max_)] is −0.70. Significant linear correlation was also found between the MAP estimates of χ and tree height (slope = 4.74 min m^−1^, *R*^2^ = 0.814, *P* < 0.0001; Figure 7).

## Discussion

The Bayesian hierarchical model performed generally well (Figure 2), and the parameter estimation was mostly robust in the cross-validation (Supplementary Figures 1A, B). The model suggests that the mid-summer stomatal conductance was usually higher in the young sample trees than in the old (Figure 4). Additionally, significant correlations were detected between tree height and (1) *A*_L_:*A*_SW_ (ρ; Figure 6) and (2) the time lag between basal sap flow and transpiration (Figure 7). Thus, the hypotheses were supported.

### Stomatal Conductance and Its Parameters

Many previous studies have reported tree age/height-related decline in stomatal conductance in several pine species, including Scots pine (e.g., Niinemets, 2002; Magnani et al., 2008; Tor-ngern et al., 2017). This decline has also been found associated with decreasing leaf area- and mass-based assimilation rate as tree height/age increased (Niinemets, 2002). Thus, declining stomatal conductance is believed to be a crucial factor underlying the declining production and growth of aged coniferous trees (Koch et al., 2004; Ryan et al., 2006; Martínez-Vilalta et al., 2007). However, the influences of VPD and irradiance on stomatal conductance of old and young trees have not been discussed as thoroughly as the generic age/size-related trends themselves. The current results on the sensitivities of *g* to *D* and *I* provide insights in this respect.

In this study, *D* and *I* showed typical daily patterns and a correlation at moderate level (Figure 1, Supplementary Table 1). Meanwhile, the near-constant ∂*g*/∂*D* in all trees since low *D* suggests that *g* in original unit was less sensitive to *D* than to *I*, to which ∂*g*/∂*I* responded throughout the measurement (Figures 5A,B). Hence, at low to medium levels of PPFD both *g*_Y_ and *g*_*O*_ were mainly influenced by irradiance. As the young trees had a higher initial slope (ι) in their IRC, *g*_Y_ increased faster than *g*_*O*_ at low to medium *I* (Equation 4, Table 3A). However, *g*_Y_−*g*_*O*_ was vanishing with increasing *I* and the relative sensitivity $C_{I}^{g}$declined faster in the young trees (Figure 5D), reflecting that their light saturation (γ) had values almost indistinguishable from each other (Table 3A). Correspondingly, the dominant factor of *g*_Y_ was shifted from *I* to *D* at a lower level of the factors than *g*_*O*_ (Figures 5C,D). Consequently, when both *D* and *I* were high, *g*_Y_ was even slightly lower than *g*_*O*_ (Figure 4). Hence, the young trees likely utilized the conditions of low to medium irradiance in their gas exchange better, whereas the old trees seemed less sensitive to close their stomata under high VPD and strong irradiance.

The higher sensitivity of stomatal control to VPD of the young trees may be due to three reasons. Firstly, previous homoeostatic analyses employing sap flow measurement have found that trees with higher stomatal conductance at low VPD are more sensitive to increasing VPD (e.g., Whitehead and Jarvis, 1981; Oren et al., 1999; McDowell et al., 2008). One simplistic theoretical explanation is that, because *E* = *g*(*D*)·*D* (Equation 4), the trees with higher *g* at low *D* (and *I*) need to decrease *g* by a larger magnitude with increasing *D* to avoid excessive transpiration (*E*) (Oren et al., 1999). Secondly, older/larger trees could be more resilient to short-time variations in *D* due to their larger internal water storage. Therefore, their sap flow is less coupled with these environmental factors, and their stomatal control is less sensitive to increasing *D* than young trees'. The third reason may lie in the environmental conditions; several daily maxima *D* were higher at the lower than the higher height in the study period (Figure 1), and the young trees' site was sub-xeric while that of the old was mesic. These drier conditions might have induced changes in water use strategies of the young trees, which would typically be age/size-related if the sample trees had been in same environmental conditions. Such changes might include more conservative gas exchange (Day and Greenwood, 2011) and higher water use efficiency (WUE) supported by the results of ^13^C-fractionation (e.g., Yoder et al., 1994; Drake et al., 2010; McDowell et al., 2011; the marginal WUE is represented by λ in the current study).

Apart from its impact on stomatal conductance, the difference between ι_Y_ and ι_*O*_ is notable as such. The initial slope of IRC has typically not been found to vary within a photosynthetic pathway (e.g., C_3_) at leaf level (Kubiske and Pregitzer, 1996; Niinemets et al., 1999; Lasslop et al., 2010; Lang et al., 2013; Jones, 2014; Mayoral et al., 2015). However, it may vary on whole-plant scale due to differences in chlorophyll concentration or nutrient allocation (Lambers et al., 2008). For instance, whole-tree GPP is positively correlated with nitrogen concentration ([N]) at low PPFD already (Peltoniemi et al., 2012a), which is against the notion that [N] only matters at high PPFD suggested by leaf-level models (e.g., Ollinger et al., 2008). Lower [N] may explain the lower ι of the old sample trees to some extent, considering that [N] is expected to decrease when conifers increase in age, size and leaf mass-area ratio (LMA) (Steppe et al., 2011). However, the evidence of this trend in Scots pine is limited from field studies (Niinemets, 2002; Martínez-Vilalta et al., 2007). The potential explanation of chlorophyll concentration also needs further clarification. Although declining chlorophyll concentration with increasing height has been reported in very tall redwood (over 50-m-tall *Sequoia sempervirens*), especially when canopy openness is low (Ishii et al., 2008; Ishii, 2011), such evidence is unknown from boreal Scots pine. Thus, the ecophysiological reasons behind ι_Y_ > ι_*O*_ are yet to be confirmed.

### Tree Height-Related Trends in Leaf-Sapwood Area Ratio and Time Lag

**Figure 8:**
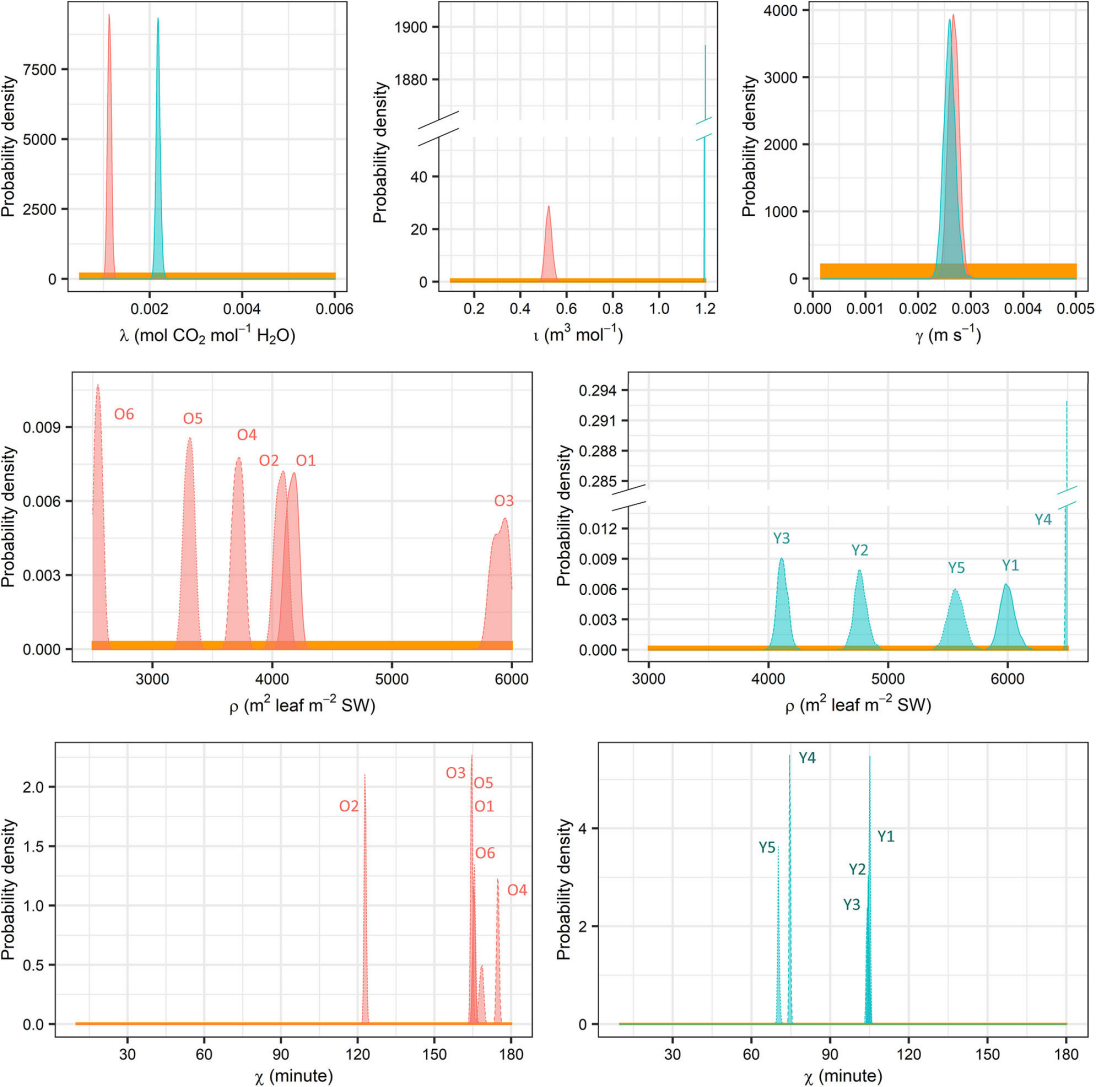
Prior (yellow) and posterior (red, old trees; cyan, young trees) distributions of λ, ι, γ, ρ, and χ. SW, sapwood.

The aforementioned major challenges in bridging transpiration and sap flow in modelling, namely the leaf-sapwood area ratio and the time lag between sap flow and transpiration, were adequately accounted for in the current study. Compared to the prior ranges given by literature, all except one (ρ_*O*3_) tree-specific ρ and χ showed reasonable MAP estimates (Figures 6–8). A significant linear correlation was detected between ρ and tree height (*H*; Figure 6), and the slope between the standardized variables (−0.70) was close to the values reported previously in the same species in a similar environment (−0.67; Vanninen et al., 1996; McDowell et al., 2002b). Amid many coniferous trees, Scots pine has shown a declining *A*_L_:*A*_SW_ (*A*_SW_ measured at breast height) with increasing age and/or size in a variety of climates and environments (e.g., Mencuccini and Grace, 1995, 1996b; Mencuccini and Bonosi, 2001; McDowell et al., 2002b; Martínez-Vilalta et al., 2007; Poyatos et al., 2007). Although consensus has yet to be reached on the mechanisms underlying this trend on large spatial scales (Poyatos et al., 2007), adjusting *A*_L_:*A*_SW_ has been widely considered as an adaptation to increased evaporative demand and a trade-off of transport efficiency for safety against embolism (Whitehead and Jarvis, 1981; Whitehead et al., 1984; Oren et al., 1999; McDowell et al., 2002b, 2008; Martínez-Vilalta et al., 2009). The current results support this conclusion. Combined with the results of *g*, we conclude that both lower *A*_L_:*A*_SW_ and stomatal conductance are features of the more conservative water use strategy in old Scots pine (McDowell et al., 2002b, 2008, 2011; Martínez-Vilalta et al., 2009; Steppe et al., 2011; Tor-ngern et al., 2017). Nevertheless, the current model structure (Equations 4, 5) and dataset were insufficient to quantitatively partition the tree age/size-related variations in *A*_L_:*A*_SW_ and stomatal conductance.

Significant correlation was also detected between χ and *H* with a slope of 4.74 min m^−1^ (Figure 7). Shown by an analogy to electricity in simplified analysis, the time lag depends on the product of hydraulic resistance and capacitance (storage), namely the time constant (τ), and in a linear dynamic model it denotes the time taken to discharge or refill 63% of the total storage (Jones, 2014). Thus, the current study suggests that hydraulic resistance and/or capacitance increases with increasing tree height, and older/taller trees need a longer time to reach another steady state of hydraulics after environmental fluctuations in mid-summer. It also suggests, in terms that $\tau \propto f_{c}^{-1}$ (where *f*_c_ denotes the cutoff frequency of low-pass filter), that the young trees could attenuate less high-frequency fluctuations in the environmental factors and thus showed less stable hydraulic dynamics than the old trees' (Figure 3). This may be a reason behind the young trees' noisier dynamics and higher RMSE. This analogy to electricity, however, is simplistic, and additional ecophysiological or technical factors include response lag of stomata, phloem flow and thermal sensitivity of the sap flow apparatus (Phillips et al., 1997, 2004; Perämäki et al., 2001; Sevanto et al., 2002; Hölttä et al., 2015). Their impacts should be clarified in the future by dynamic modelling on the time lag (cf. e.g., Phillips et al., 1997, 2004; Bell et al., 2015).

### Merits and Limits of the Model

The current hierarchical model directly bridges sap flow and stomatal behaviour, and thus enables prediction of whole-tree hydraulic dynamics, including the estimation of *A*_L_:*A*_SW_ and the time lag between the influx (sap flow) and efflux (transpiration), in a cohesive manner (Equations 5–8). Compared with earlier studies by Bayesian analysis on trees' hydraulics with empirical canopy conductance models (e.g., Samanta et al., 2008), the current model employed a more process-based structure (Equation 4) on stomatal behaviour. By this feature, the inverse method in the current study disentangled tree age/size-related effects on whole-tree hydraulic dynamics to some extent. The input data, VPD, PPFD and sap flow density, are widely accessible or measurable with low technical difficulty. The overall performance was good, but tree-specific errors differed considerably (Figures 2, 3). However, most of the uncertainties were due to measurement error, while the parametric uncertainty was very small (Figure 3). Meanwhile, most of the parameter estimates seemed robust although those of γ_Y_, χ_*Y*4_ and χ_*Y*5_ shifted in several occasions during the cross-validation (Supplementary Figures 1A,B). This indicates a fairly reliable parameterization with potential for better performance with improved measurements of sap flow density and/or environmental factors.

Besides the impacts of the smaller time constant of young trees, the noticeably higher uncertainties and measurement error in the young trees may be mainly due to their more open canopy and related to the lesser robustness of γ_Y_, χ_*Y*4_ and χ_*Y*5_. Their canopy structure occasionally allowed direct sunshine on the probes despite the shading aluminium foil. This should have caused extremely high values of *J*^(O)^ with high error because of the shifted temperature gradient in the tree trunk and the transported water (Köstner et al., 1998). Such values should have particularly influenced the estimation of γ as it is the asymptote of the IRC (Equation 4). Higher measurement errors in the meteorological and/or sap flow data were also likely caused by the weather events bringing about low *D* and *I* during daytime (e.g., the rainfall on DOY 202 during the measurement on trees O2—O6; Figure 3). Yet another possible source of measurement error is the thermal sensitivity of the probes, which may have particularly influenced the estimation of χ (Hölttä et al., 2015). However, this artefact, if it occurred, should be systematic and could not explain the linear correlation between χ and *H*.

The total number of parameters was large (44), but the risk of over-parameterization was low. The first reason is that only 28 parameters (λ_*AG*_, ι_*AG*_, γ_*AG*_, ρ_*m*_, χ_*m*_) were employed directly for simulation, whereas the other 16 (*a*_*m*_Y__, *b*_*m*_Y__, *c*_*AG*_, μ_*AG*_, σ_*AG*_; Supplementary Tables 2B, 3B) were to define the distributions of other parameters or ε. Moreover, the 28 parameters were either age group- or tree-specific, resulting in only five being directly used in the prediction of each sample tree. This allowed a high degree of freedom due to the sizeable data set of one tree (at least 882 valid observations). The main role of *a*_*m*_Y__, *b*_*m*_Y__, *c*_*AG*_, μ_*AG*_ and σ_*AG*_ was regulating the disturbance from outlying observations, if there was any, judged by prior knowledge (Table 2), while they did not reduce the degree of freedom in simulation. The utilization of prior knowledge is a generic advantage of the Bayesian inference, and it allows future studies to treat the current one as prior knowledge and efficiently update for their needs. The Bayesian framework also enabled uncertainty partitioning (Figure 3), which helped draw conclusions on the reliability of parameter estimation and data quality.

The following warnings on the model design should be emphasized. Firstly, the linear structure of *J, g*, and ρ failed to partition quantitatively the tree age/size-induced effects on the latter two, albeit their modelled values or MAP estimates generally fell well into the prior ranges given by literature. Secondly, one single set of parameters were applied to one sample tree without accounting for the heterogeneity inside the canopy. This “big-leaf assumption” excludes direct comparison of the parameter estimates with the previous studies that employed similar models as the latter focussed on individual shoots (e.g., Hari and Mäkelä, 2003; Mäkelä et al., 2004). Also, canopy gaps, which were present at the young site in the current study, may result in a poorer performance of the Bayesian models based on the big-leaf assumption (Mackay et al., 2012). However, the tree-specific (rather than stand-specific) parameters in the current study may have mitigated this drawback to some extent. Thirdly, the time lag may differ between water storage discharge and refilling or between the increases and decreases of *D* and *I*, because hydraulic resistance and/or capacitance in trees may be dependent on the internal water content (Tyree and Zimmermann, 2002) rather than being constant. Additionally, the current study did not include a minimum limit of *g*, which would reflect stomatal leakage and cuticular conductance and is common in precedent works [e.g., Hari and Mäkelä, 2003; Mäkelä et al., 2004; and see e.g., Tuzet et al. (2003) and Peltoniemi et al. (2012b) for a similar design in different model structure]. It was not applied in this study because of the lack of experimental support on quantification in Scots pine. Finally, the MAP estimate of ι_Y_ fell on its initial maximum (Figure 8, Supplementary Table 3B), but we decided not to expand its initial range considering the physiological meaning and correlation with other parameters (Supplementary Table 2B, Supplementary Figure 2).

As we focussed on modelling hydraulic dynamics on the temporal scale of minutes, the applicability of this approach for longer-term studies should be treated with caution. Firstly, the assumption that the sums of whole-tree transpiration and sap flow equal over the study period, i.e. *A*_*SW*_∫*J*(*t*)*dt* = *A*_*L*_∫*E*(*t*)*dt*, which allowed the linear *J* = ρ*E* (Equation 5), may fail due to changes in water content in tree canopy and/or soil on larger temporal scales. Also, the marginal water use efficiency (λ) may vary across time in long term, and thus a single estimate per tree should entail larger uncertainties than in the current study. Lastly, the non-process-based modelling of time lag limits its applicability when the physiological processes or traits (e.g., discharge and recharge of hydraulic capacitance) become time-variant on larger temporal scales.

### Summary

By Bayesian hierarchical modelling, we bridged an optimization model of stomatal behaviour and sap flow measurement to simulate whole-tree hydraulic dynamics of Scots pine in mid-summer in southern Finland. The model's overall performance was good, although tree-specifically it was limited mainly by measurement error in the young trees. According to the model, stomatal conductance was usually higher in the young than the old trees, but the difference was very small at high VPD and PPFD. The results suggest that the young trees' gas exchange was more activated by low to medium PPFD and is slightly more sensitive to increasing VPD. The tree age-related weaker response to PPFD may be due to decreased nitrogen and/or chlorophyll concentrations. The young trees' slightly higher sensitivity to VPD was possibly impacted by the drier site conditions, but also accords with the larger internal water storage and/or lower leaf-sapwood area ratio (*A*_L_:*A*_SW_, *A*_SW_ at breast height) found in old trees. *A*_L_:*A*_SW_ and time lag were both well-correlated with tree height. The decrease in *A*_L_:*A*_SW_ with tree height was likely to avoid excessive transpiration and embolism, and time lag increased with tree height due to longer transport, higher whole-tree hydraulic resistance and/or capacitance. Future work is expected in reducing measurement error, modelling in-crown heterogeneity of environmental factors and tree ecophysiological activities, and quantifying time lag by means of dynamic analyses.

## Data Availability Statement

The raw data supporting the conclusions of this article will be made available by the authors, without undue reservation.

## Author Contributions

CL, TH, and AM designed the research. CL conducted fieldwork with FB advice. CL, TH, XT, and AM analysed data. CL wrote and TH, XT, FB, and AM edited the manuscript. All authors contributed to the article and approved the submitted version.

## Conflict of Interest

The authors declare that the research was conducted in the absence of any commercial or financial relationships that could be construed as a potential conflict of interest.
