# Supplementary material for: Weaker Light Response, Lower Stomatal Conductance and Structural Changes in Old Boreal Conifers Implied by a Bayesian Hierarchical Model
Source: Front Plant Sci. 2020 Nov 6;11:579319. doi: 10.3389/fpls.2020.579319 (PMC7677260; doi:10.3389/fpls.2020.579319)
Supplement: Supplementary file 1 [file Table_1.DOCX]

Supplementary Material

This Supplementary Material includes one textual section (358 words, excluding References), three tables, and two figures in colour (in a separate file).

# Whole-cross-section sap flow density

During the setup of sap flow measurement, the locations of the probes relative to a circumference of reference nails on the trunk were recorded with callipers, and the central angles of sectors in the four ordinal directions (*ω_k_*, where *k* denotes the direction) were calculated. This appendix depicts the calculation from the raw sap flow density (*J*, **Error! Reference source not found.**) to whole-tree sap flow density (*J_m_*). The sap flow density in each direction ($\bar{J_{k}}$) depended on the relationship between probe length (*l*_p_) and sapwood depth ($\vartheta_{\mathrm{SW}}$). If $\vartheta_{\mathrm{SW}}$ > *l*_p_, $\bar{J_{k}}$ was scaled by averaging the estimated whole-sector sap flow rate ($\bar{Q}$; m^3^ s^-1^) over the sapwood area of the sector (*A*_SW_; m^-2^). $\bar{Q}$ was estimated by the gamma distribution in relation to the depth into sapwood (Berdanier et al. 2016) as

|  | $\bar{Q}=2\pi J\int_{0}^{r} \left( r-\vartheta\right)\left( \beta\vartheta\right)^{\alpha}e^{-\beta\vartheta}d\vartheta$ | **Eqn S1** |
| --- | --- | --- |

where *r* is the radius of tree trunk at the probe, $\vartheta$ the depth to cambium, and *α* and *β* are parameters; and

|  | $A_{\mathrm{SW}}=\pi r^{2}-\pi{(r-\vartheta_{S})}^{2}$ | **Eqn S2** |
| --- | --- | --- |

(both as if applicable to the whole cross section of trunk). Thus,

|  | $\bar{J_{k}}=\frac{\bar{Q}}{A_{\mathrm{SW}}}=\frac{2 J\int_{0}^{r} \left( r-\vartheta\right)\left( \beta\vartheta\right)^{\alpha}e^{-\beta\vartheta}d\vartheta}{r^{2}-{(r-\vartheta_{S})}^{2}}$ | **Eqn S3** |
| --- | --- | --- |

If $\vartheta_{\mathrm{SW}}$ < *l*_p_, the recorded Δ*U* was adjusted by the proportions of sapwood (noted Δ*U*_SW_) and the non-conducting heartwood (Δ*U*_HW_) by assuming heartwood had the same thermal properties as sapwood at *J* = 0 (i.e. Δ*U*_HW_ ≡ Δ*U^*^*; Clearwater et al. 1999; Lu et al. 2004). This is to say

|  | $\Delta U=\beta_{\mathrm{SW}}\Delta U_{\mathrm{SW}}+\beta_{\mathrm{HW}}\Delta U_{\mathrm{HW}}$ |  |
| --- | --- | --- |

and thus

|  | $\Delta U_{\mathrm{SW}}=\frac{\Delta U-\beta_{\mathrm{HW}}\Delta U^{*}}{\beta_{\mathrm{SW}}}$ | **Eqn S4** |
| --- | --- | --- |

where $\beta_{\mathrm{SW}}={\vartheta_{\mathrm{SW}}}/{l_{p}}$ and $\beta_{\mathrm{HW}}=1-\beta_{\mathrm{SW}}$. Substituting Δ*U*_SW_ in Eqn S4 with Δ*U* in **Error! Reference source not found.**, the whole-sector sap flow density (as if applicable to the whole cross section of trunk) is

|  | $\bar{J_{k}}=118.99\times{10}^{-6}\left( \frac{\beta_{\mathrm{SW}}}{\frac{\Delta U}{\Delta U^{*}}-\beta_{\mathrm{HW}}}-1 \right)^{1.231}$ | **Eqn S5** |
| --- | --- | --- |

The mean of $\bar{J_{k}}$ (computed by either Eqn S3 or Eqn S5) weighted by *ω_k_* was defined as the whole-cross-section sap flow density (i.e. $J_{m}^{\left( O \right)}$ used in the data model [Eqns 6 and 7], where *m* denotes the ordinal numbers of trees as in Table 1). It was then converted to mol m^-2^ SW s^-1^ to comply with the process model (Eqn 4). Hence,

|  | $J_{m}^{\left( O \right)}=\frac{\sum_{k} \bar{J_{k}}\omega_{k}}{2\pi}\times\frac{\rho_{H_{2}O}}{M_{H_{2}O}}$ | **Eqn S6** |
| --- | --- | --- |

where the density and molar mass of water, respectively, are $\rho_{H_{2}O}$ = 1 × 10^3^ kg m^-3^ and $M_{H_{2}O}$ = 18.01528 × 10^-3^ kg mol^-1^.

## References

Berdanier, A. B., Miniat, C. F., and Clark, J. S. (2016). Predictive models for radial sap flux variation in coniferous, diffuse-porous and ring-porous temperate trees. Tree Physiol. 36(8), 932-941. doi: [10.1093/treephys/tpw027](https://doi.org/10.1093/treephys/tpw027)

Clearwater, M. J., Meinzer, F. C., Andrade, J. L., Goldstein, G., and Holbrook, N. M. (1999). Potential errors in measurement of nonuniform sap flow using heat dissipation probes. Tree Physiol. 19(10), 681-687. doi: 10.1093/treephys/19.10.681

Lu, P., Urban, L., and Zhao, P. (2004). Granier's thermal dissipation probe (TDP) method for measuring sap flow in trees: theory and practice. Acta Botanica Sinica (English Edition) 46(6), 631-646.

# Supplementary Tables and Figures

## Supplementary Tables

**Table S1** Pairwise Pearson (upper-right) and Spearman (lower-left) correlation coefficients between D at the higher (D_high_) and the lower heights (D_low_) and I.

|  | *D*_high_ | *D*_low_ | *I* |
| --- | --- | --- | --- |
| *D*_high_ |  | 0.995 | 0.459 |
| *D*_low_ | 0.994 |  | 0.500 |
| *I* | 0.461 | 0.504 |  |

All data are non-zero. All *P* < 0.0001.

**Table S2** List of symbols used for **(A)** data processing and **(B)** Bayesian hierarchical modelling.

**(A)**

| Symbol | Type^*^ | Meaning | Value and typical unit^§^ |
| --- | --- | --- | --- |
| *J* | V | Sap flow density | mol m^-2^ SW s^-1^ |
| *D* | V | Vapour deficit | mol m^-3^ |
| *I* | V | Photosynthetic photon flux rate (PPFD) | mol m^-2^ leaf s^-1^ |
| (Δ)*U* | V | Voltage (and difference) | mV |
| VPD | V | Vapour pressure deficit | Pa |
| *Q* | V | Sap flow rate | m^3^ s^-1^ |
| *A* | V | Area | m^2^ |
| $\vartheta$ | V | Depth (e.g. of sapwood) | m |
| *r* | V | Radius of tree trunk | m |
| *ω* | V | Central angle of measured sector of tree trunk | (rad) |
| *T* | V | Air temperature | °C |
| *h_r_* | V | Relative humidity | (%) |
| *α, β* | P | Shape (*α*) and rate (*β*) parameters of the gamma distribution of sap flow density on cross section of trunk | -- |
| *β*_□_ | P | Proportions of sapwood and heartwood (see subscript below) to probe length provided the probe entered heartwood | -- |
| *k* | S | Ordinal direction of a sector of sample tree trunk | -- |
| $\rho_{H_{2}O}$ | C | Density of water | 1000 kg m^-3^ |
| $M_{H_{2}O}$ | C | Molar mass of water | 1.801528 × 10^-2^ kg mol^-1^ |
| *P*_0_ | C | Standard atmospheric pressure | 1.01325 × 10^5^ Pa |
| *R* | C | Ideal gas constant | 8.3145 J K^-1^ mol^-1^ |

**(B)**

| Symbol | Type^*^ | Meaning | Value and typical unit^§^ |
| --- | --- | --- | --- |
| *J* | V | (See **(A)**) | (See **(A)**) |
| *D, I* | V, S |  |  |
| *g* | V, S | Stomatal conductance | m s^-1^ |
| *E* | V | Transpiration | mol m^-2^ leaf s^-1^ |
| *ε* | V | Error term, difference between modelled and measured sap flow density | mol m^-2^ SW s^-1^ |
| *C* | V | Dimensionless sensitivity coefficient | -- |
| *λ* | P | Marginal carbon gain per water cost | mol CO_2_ mol^-1^ H_2_O |
| *ι* | P | Initial slope of PPFD response curve | m^3^ mol^-1^ |
| *γ* | P | Saturation level (asymptote) of PPFD response curve | m s^-1^ |
| *ρ* | P | (All-sided) Leaf-sapwood areas ratio | m^2^ leaf m^-2^ SW |
| *μ, σ* | P | Mean (*μ*) and variance (*σ*) of the heavy-tailed normal distribution of leaf-sapwood areas ratios | -- |
| *a, b, c* | P | For expressing the rate parameter of the Laplace distribution of the error between modelled and measured sap flow density | -- |
| *χ* | P, S | Elapsed time between modelled transpiration and measured sap flow density | minute |
| ***θ*** | P | Parameter vector of the process and data models, i.e. *λ, ι, γ, ρ* and *χ*. | (Various) |
| *t* | S | Time | minute |
| *m* | S | Ordinal number of tree (see Table 1) | -- |
| AG / Y, O | S | Age group, including Y = young and O = old (see Table 1) | -- |
| (M), (O) | S | Modelled (M) and measured (observed; O) (sap flow density) | -- |
| *C*_a_ | C | CO_2_ concentration in the atmosphere | 1.701 × 10^-2^ mol m^-3^ |

^*^V, variable; P, parameter; S, super- or subscript; C, constant.

^§^Only the values of constants are given; SW, sapwood.

**Table S3** **(A)** Prior ranges and **(B)** maxima a posteriori (MAP) estimates with 95% Bayesian credible intervals of the calibrated parameters.

**(A)**

| Parameter |  | Prior range | |
| --- | --- | --- | --- |
|  |  | Min. | Max. |
| $\lambda_{Y}$, $\lambda_{O}$ |  | 0.5 × 10^-3^ | 6.0 × 10^-3^ |
| $\iota_{Y}$, $\iota_{O}$ |  | 0.1 | 1.2 |
| $\gamma_{Y}$, $\gamma_{O}$ |  | 1.6 × 10^-3^ | 5.0 × 10^-3^ |
| $\chi_{Y}$, $\chi_{O}$ | * | 10 | 180 |
| $\rho_{Y}$ | * | 3000 | 6500 |
| $\rho_{O}$ | * | 2500 | 6000 |
| $a_{Y}$ | * | 0 | 5 |
| $b_{Y}$ | * | 0 | 12 |
| $c_{Y}$, $c_{O}$ |  | 1 × 10^-4^ | 3 |
| $\mu_{Y}$ |  | 3000 | 6500 |
| $\mu_{O}$ |  | 2500 | 6000 |
| $\sigma_{Y}$ |  | 52.28 | 522.82 |
| $\sigma_{O}$ |  | 32.72 | 327.22 |

*Tree-specific during estimation

See Table S2(B) for the parameters’ meanings and units.

**(B)**

| Parameter | MAP | 2.5% | 97.5% | Parameter | MAP | 2.5% | | 97.5% |
| --- | --- | --- | --- | --- | --- | --- | --- | --- |
| $\lambda_{Y}$ | 2.182  × 10^-3^ | 2.103  × 10^-3^ | 2.277  × 10^-3^ | $\rho_{Y1}$ | 6003 | | 5885 | 6121 |
| $\lambda_{O}$ | 1.124  × 10^-3^ | 1.056  × 10^-3^ | 1.211  × 10^-3^ | $\rho_{Y2}$ | 4774 | | 4670 | 4880 |
| $\iota_{Y}$ | 1.200 | 1.197 | 1.200 | $\rho_{Y3}$ | 4100 | | 4037 | 4201 |
| $\iota_{O}$ | 0.520 | 0.497 | 0.550 | $\rho_{Y4}$ | 6493 | | 6486 | 6500 |
| $\gamma_{Y}$ | 2.594  × 10^-3^ | 2.400  × 10^-3^ | 2.816  × 10^-3^ | $\rho_{Y5}$ | 5551 | | 5441 | 5696 |
| $\gamma_{O}$ | 2.625  × 10^-3^ | 2.500  × 10^-3^ | 2.869  × 10^-3^ | $\rho_{O1}$ | 4172 | | 4080 | 4237 |
| $a_{Y2}$ | 0.001 | 0.000 | 0.004 | $\rho_{O2}$ | 4084 | | 3992 | 4149 |
| $a_{Y3}$ | 0.021 | 0.008 | 0.072 | $\rho_{O3}$ | 5926 | | 5786 | 5994 |
| $a_{Y4}$ | 0.078 | 0.037 | 0.116 | $\rho_{O4}$ | 3728 | | 3639 | 3781 |
| $a_{Y5}$ | 4.907 | 3.563 | 4.988 | $\rho_{O5}$ | 3317 | | 3239 | 3368 |
| $a_{Y6}$ | 4.969 | 4.113 | 4.994 | $\rho_{O6}$ | 2564 | | 2503 | 2593 |
| $b_{Y2}$ | 0.953 | 0.136 | 11.561 | $\chi_{Y2}$ | 104.5 | | 104.2 | 105.3 |
| $b_{Y3}$ | 0.473 | 0.097 | 10.946 | $\chi_{Y3}$ | 105.1 | | 104.6 | 105.4 |
| $b_{Y4}$ | 0.287 | 0.182 | 0.537 | $\chi_{Y4}$ | 104.2 | | 103.6 | 104.8 |
| $b_{Y5}$ | 0.097 | 0.068 | 0.120 | $\chi_{Y5}$ | 74.3 | | 74.3 | 75.1 |
| $b_{Y6}$ | 0.124 | 0.092 | 0.140 | $\chi_{Y6}$ | 70.4 | | 70.0 | 71.1 |
| $c_{Y}$ | 0.932 | 0.926 | 0.954 | $\chi_{O1}$ | 165.8 | | 164.5 | 166.3 |
| $c_{O}$ | 0.781 | 0.767 | 0.788 | $\chi_{O2}$ | 122.7 | | 122.2 | 123.7 |
| $\mu_{Y}$ | 5488 | 4559 | 6266 | $\chi_{O3}$ | 164.7 | | 163.9 | 165.3 |
| $\mu_{O}$ | 3958 | 3248 | 4287 | $\chi_{O4}$ | 174.7 | | 173.9 | 175.7 |
| $\sigma_{Y}$ | 297 | 170 | 516 | $\chi_{O5}$ | 165.5 | | 164.8 | 166.7 |
| $\sigma_{O}$ | 210 | 124 | 324 | $\chi_{O6}$ | 168.5 | | 166.7 | 169.7 |

See Table S2(B) for the parameters’ meanings and units.
