# Supplementary material for: Weaker Light Response, Lower Stomatal Conductance and Structural Changes in Old Boreal Conifers Implied by a Bayesian Hierarchical Model
Source: Front Plant Sci. 2020 Nov 6;11:579319. doi: 10.3389/fpls.2020.579319 (PMC7677260; doi:10.3389/fpls.2020.579319)
Supplement: Supplementary file 2 [file Image_1.PDF]

2.2 Supplementary Figures

(A)

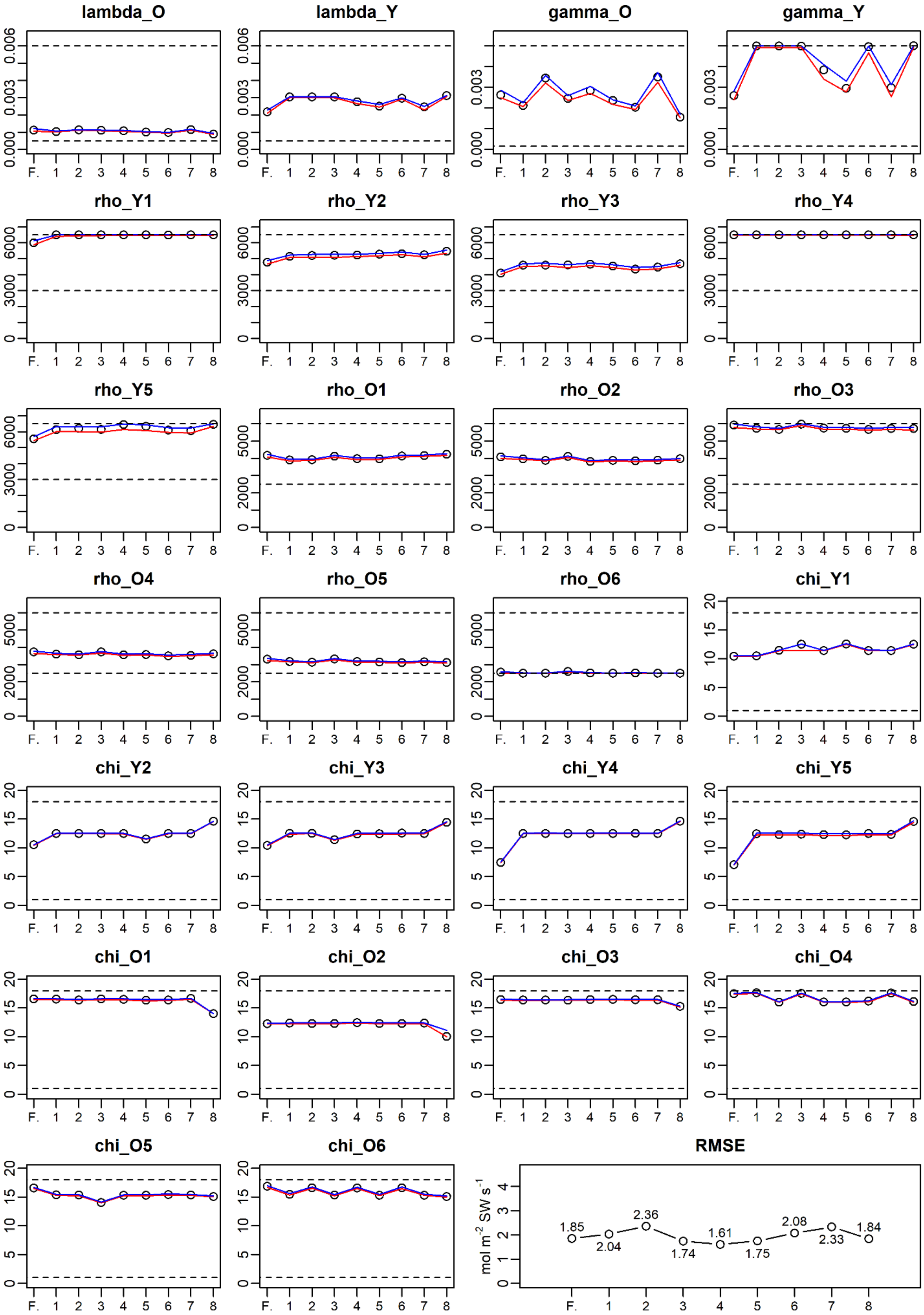

(B)

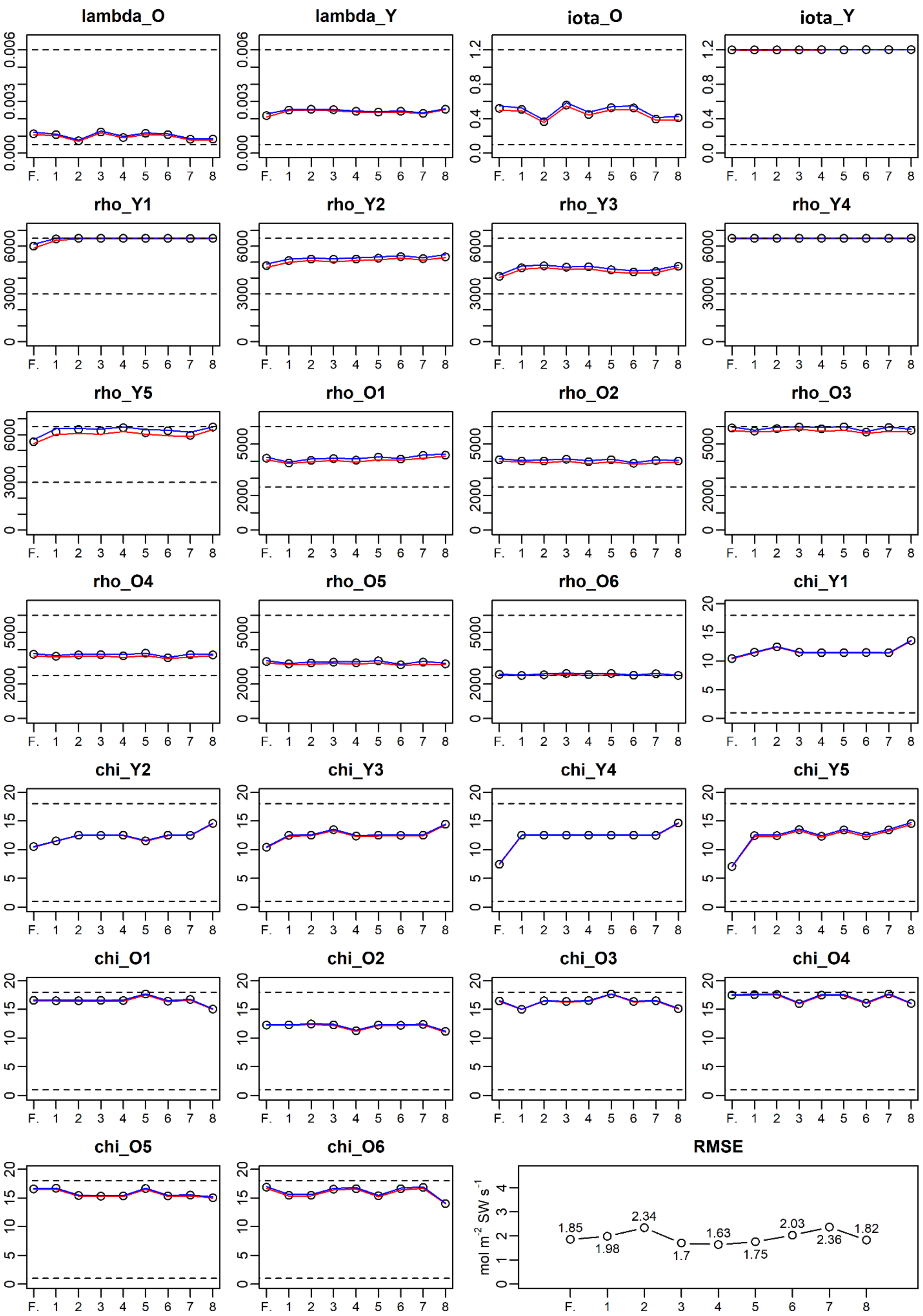

Figure S1 Results of the eight-fold cross-validation with **(A)** fixed ι_Y and ι_O and **(B)** fixed γ_Y and γ_O in comparison to those with full data (F.). In parameter estimation, the maxima a posteriori

(MAP) estimates (circle) and 95% credible intervals (delimited by red and blue lines) are shown with the initial range (dashed lines). The Greek letters in the parameters' names are spelt out; see Table S2(A,B) for their units. RMSE, root-mean-square error.

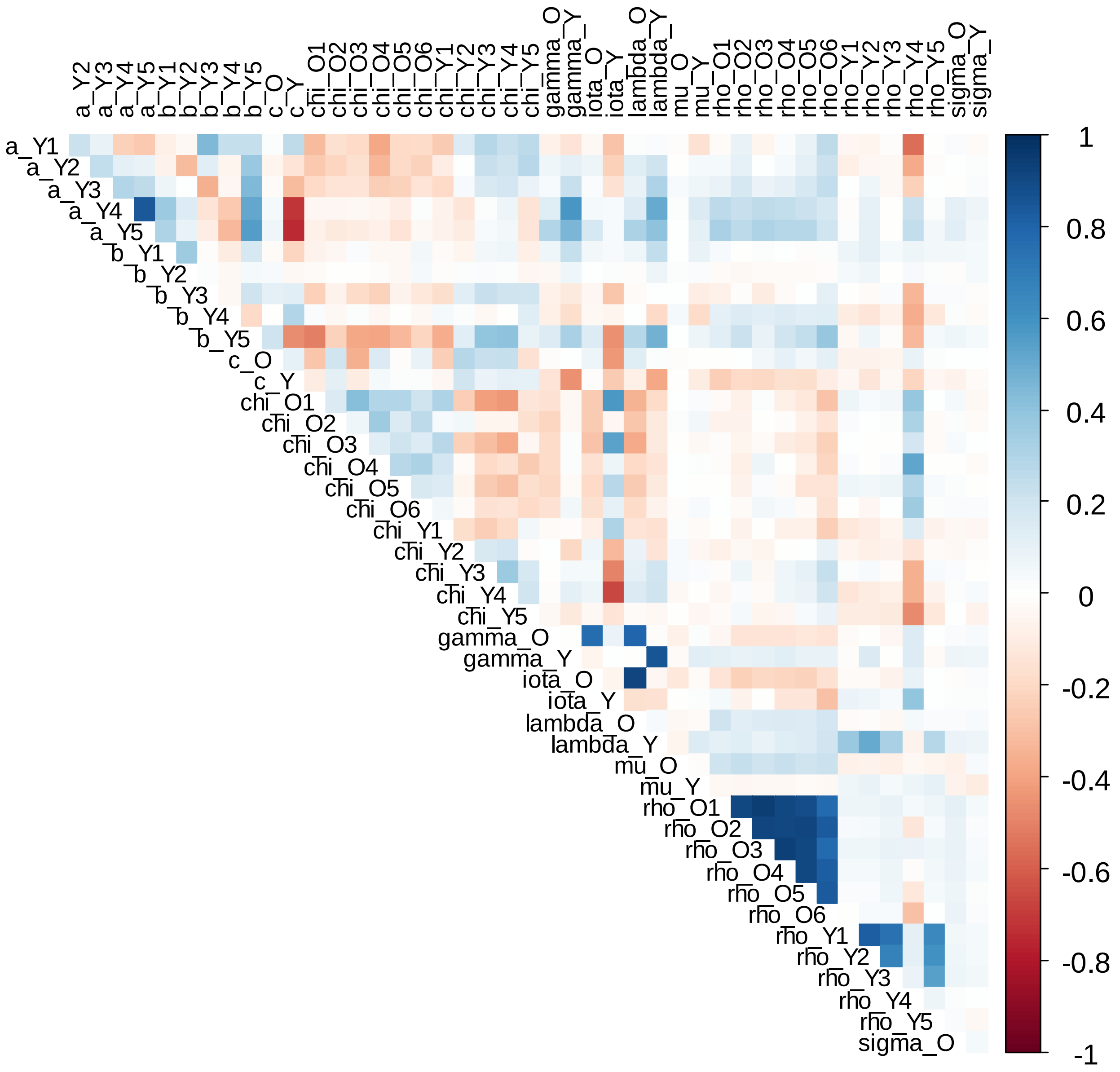

Fig. S2 Correlation matrix of the calibrated parameters. See Table S2(B) for their definitions and units. The Greek letters in the parameters' names are spelt out. The colour legend of the correlation coefficient is on the right.
